# GazeCapsNet: A Lightweight Gaze Estimation Framework

**DOI:** 10.3390/s25041224

**Published:** 2025-02-17

**Authors:** Shakhnoza Muksimova, Yakhyokhuja Valikhujaev, Sabina Umirzakova, Jushkin Baltayev, Young Im Cho

**Affiliations:** 1 Department of Computer Engineering, Gachon University, Sujeong-gu, Seongnam-si 461-701, Republic of Korea; shakhnoza02@gachon.ac.kr; 2 Aria Studios Co., Ltd., Seoul 06247, Republic of Korea; yakhyo9696@gmail.com; 3 Department of Information Systems and Technologies of the Tashkent State University of Economic, Tashkent 100066, Uzbekistan; j_baltayev@tsue.uz

**Keywords:** eye appearance, capsule networks, self-attention routing mechanism, lightweight architectures, gaze estimation

## Abstract

Gaze estimation is increasingly pivotal in applications spanning virtual reality, augmented reality, and driver monitoring systems, necessitating efficient yet accurate models for mobile deployment. Current methodologies often fall short, particularly in mobile settings, due to their extensive computational requirements or reliance on intricate pre-processing. Addressing these limitations, we present Mobile-GazeCapsNet, an innovative gaze estimation framework that harnesses the strengths of capsule networks and integrates them with lightweight architectures such as MobileNet v2, MobileOne, and ResNet-18. This framework not only eliminates the need for facial landmark detection but also significantly enhances real-time operability on mobile devices. Through the innovative use of Self-Attention Routing, GazeCapsNet dynamically allocates computational resources, thereby improving both accuracy and efficiency. Our results demonstrate that GazeCapsNet achieves competitive performance by optimizing capsule networks for gaze estimation through Self-Attention Routing (SAR), which replaces iterative routing with a lightweight attention-based mechanism, improving computational efficiency. Our results show that GazeCapsNet achieves state-of-the-art (SOTA) performance on several benchmark datasets, including ETH-XGaze and Gaze360, achieving a mean angular error (MAE) reduction of up to 15% compared to existing models. Furthermore, the model maintains a real-time processing capability of 20 milliseconds per frame while requiring only 11.7 million parameters, making it exceptionally suitable for real-time applications in resource-constrained environments. These findings not only underscore the efficacy and practicality of GazeCapsNet but also establish a new standard for mobile gaze estimation technologies.

## 1. Introduction

Estimating gaze from facial images is crucial for understanding human cognition and behavior. Currently, gaze estimation plays a vital role across various fields such as virtual reality (VR) [1,2], human–computer interaction [3,4], semi-autonomous driving [5,6], and psychological studies [7]. Paper [8] presents a mobile gaze estimation framework that utilizes a real-time algorithm operating at 30 Hz. It predicts eye movements from sequential frames with high accuracy and fewer parameters, enhancing AR/VR interactions. Achieving real-time performance in mobile environments poses significant challenges due to the limited processing capabilities of these platforms. Traditional convolutional neural networks (CNNs), while robust in various visual recognition tasks [9], often struggle to capture the complex spatial hierarchies essential for accurate gaze prediction [10]. While MobileNet v2 and ResNet-18 have previously been combined for various vision tasks [11], their integration has not been extensively explored for gaze estimation. Previous studies utilizing this combination primarily focus on image classification [12] and object detection [13], where feature extraction is performed at fixed spatial scales without considering hierarchical facial feature relationships. In contrast, our GazeCapsNet framework leverages this hybrid feature extraction strategy within a capsule network-based architecture, where SAR dynamically prioritizes relevant gaze-related regions. This allows our model to retain spatial dependencies across different head poses and lighting conditions, improving generalization in real-time gaze-tracking applications.

At the core of our approach is the use of capsule networks (CapsNets) [14] with Self-Attention Routing (SAR). CapsNets address some limitations of traditional CNNs by preserving spatial hierarchies between features, which is crucial for tasks like gaze estimation. The SAR mechanism further enhances the model’s efficiency by focusing computational resources on the most relevant features for accurate gaze prediction, adapting dynamically to various real-world conditions. This study rigorously evaluates GazeCapsNet across multiple benchmark datasets, including ETH-XGaze, Gaze360, and MPIIFaceGaze, which feature various head poses, lighting conditions, and gaze directions. These datasets provide a comprehensive platform for demonstrating the robustness of our model in both controlled and uncontrolled environments.

Our work presents GazeCapsNet, a novel, lightweight deep learning framework for real-time gaze estimation. The key contributions of this study are as follows:

- We introduce a capsule network-based architecture with SAR, which enhances gaze estimation accuracy while reducing computational overhead.
- By integrating MobileNet v2 and ResNet-18, we achieve a balance between low-latency inference and robust feature extraction, enabling real-time performance.
- Unlike traditional methods that rely on intermediate steps like facial landmark detection, GazeCapsNet predicts 3D gaze direction directly from raw images, improving efficiency and generalization.
- With an inference time of 20 ms per frame and only 11.7 M parameters, our model is suitable for resource-constrained environments such as AR/VR and driver monitoring systems.
- Extensive evaluations on benchmark datasets (ETH-XGaze, Gaze360, MPIIFaceGaze) demonstrate that GazeCapsNet achieves competitive accuracy while maintaining a lightweight architecture.

Our work introduces a highly efficient, real-time gaze estimation model that integrates capsule networks with Self-Attention Routing, optimized for mobile and resource-constrained devices. We provide a comprehensive solution that balances high accuracy, scalability, and deployment efficiency, setting a new standard for gaze estimation in both controlled and real-world environments.

The remainder of this paper is organized as follows: Section 2 reviews existing advancements in lightweight mobile models and the integration of capsule networks for gaze estimation. Section 3, the methodology section, delves into the architecture of the proposed Mobile-GazeCapsNet, detailing its innovative features such as multi-scale feature aggregation and boundary refinement techniques. In Section 4, the paper describes the experimental setup, including the datasets used, and evaluation metrics, and presents the performance results, comparing them to current state-of-the-art models. Section 5, the Discussion and Limitations Section, evaluates the outcomes, acknowledges the model constraints, and suggests directions for future research. Finally, Section 6, the conclusion, summarizes the contributions of the study and its implications for real-time applications in gaze estimation.

## 2. Related Work

Traditional gaze estimation methods typically rely on CNNs for feature extraction, which are then followed by regression or classification models to predict gaze direction. Recent advancements in deep learning have led to the adoption of more sophisticated architectures such as ResNet and DenseNet. These models improve gaze prediction accuracy but are associated with significant computational costs. Capsule networks, introduced in [14], have shown promise in accurately representing spatial hierarchies of image features, making them particularly suitable for gaze estimation tasks. Other promising models such as MobileNet v2 [15] and MobileOne [16] are prominent mobile-optimized architectures, designed to reduce computation and memory usage effectively. MobileNet v2 utilizes inverted residuals and linear bottlenecks to minimize computational overhead, while MobileOne is tailored to enhance real-time inference with minimal latency. These architectures enable the efficient performance of complex vision tasks, such as gaze estimation, on mobile devices.

GazeCaps [17] introduced the use of capsule networks for gaze estimation. The model employs an SAR mechanism that dynamically allocates attention across various regions of the face, effectively handling nonlinear transformations due to head poses or lighting changes. This approach surpasses traditional CNN-based models in terms of accuracy and generalization across datasets. Recent innovations in mobile gaze estimation have incorporated novel deep learning architectures and lightweight models tailored for mobile devices. Recent studies have emphasized the emergence of mobile gaze estimation systems that leverage both hardware optimizations and advanced machine learning techniques. For instance, ref. [18] presented a method that requires minimal user interaction for calibration and demonstrated its efficacy on mobile devices through efficient algorithm design. Similarly, ref. [19] developed a gaze estimation framework that adapts dynamically to various user environments by adjusting its parameters.

Deep learning continues to significantly enhance gaze estimation accuracy. Notably, ref. [20] introduced a multi-task learning framework that simultaneously predicts eye landmarks and gaze direction, considerably reducing the error in cross-dataset evaluations. This strategy has proven to be effective in improving the model’s generalization capabilities across diverse populations and settings. Ref. [21] discussed the deployment of quantized neural networks to substantially reduce computational requirements while maintaining high accuracy, essential for real-time applications on mobile platforms. Researchers [22] explored the integration of gaze estimation into daily mobile applications, showcasing practical deployment scenarios and user interaction models. The use of edge computing in gaze tracking was pioneered by the authors of [23], which proved the feasibility of processing gaze data directly on edge devices, thereby reducing latency and enhancing responsiveness. This method is particularly advantageous for interactive systems like augmented reality, where rapid processing is paramount.

Recent studies, such as [24], have concentrated on the robustness of gaze estimation systems in natural settings. Their research includes developing algorithms capable of adapting to outdoor lighting and complex background variations, challenges that traditional systems often face. Generative Adversarial Networks (GANs) have been utilized to augment gaze estimation datasets, as noted in [25]. This technique enhances the diversity and volume of training data, crucial for improving the model’s accuracy and robustness. The integration of Recurrent Neural Networks (RNNs) with CNNs in [26] captures temporal dependencies in video-based gaze estimation tasks, a critical factor for understanding dynamic gaze shifts in real-time video streams. Transfer learning has been increasingly applied to gaze estimation, as discussed in [27], utilizing pre-trained models on large image datasets to bootstrap gaze estimation models, significantly reducing the required amount of gaze-specific data. Beyond conventional models, the use of attention mechanisms to selectively focus on the most relevant parts of the image for gaze prediction has been explored by the authors of [28]; their findings suggest that attention improves model interpretability and efficiency by reducing the influence of noisy or irrelevant data.

Application-specific adaptations, such as those for driver monitoring systems, have been developed by the authors of [29], who tailored gaze estimation models to assess driver alertness and gaze direction within the context of automotive safety. Comprehensive benchmarking of gaze estimation techniques, particularly in unconstrained environments, was performed by the authors of [30], who provided insights into the performance variations across different settings and datasets. The work in [31,32] addresses the ethical and privacy implications of gaze-tracking technologies, especially in public and semi-public spaces, highlighting the need for guidelines and regulations to govern the use of such sensitive biometric data.

Prior work in gaze estimation has primarily relied on convolutional neural networks (CNNs) for feature extraction, often coupled with additional modules such as recurrent layers or attention mechanisms to refine predictions. Some classic models are shown in Table 1. While CNN-based models like FullFace [33] employ a full-face representation for gaze prediction, improving robustness but at the cost of high inference time (50 ms per frame) and large model size (196.6 M parameters), RT-GENE [34] focuses on real-time applications but still requires 40 ms per frame, limiting its deployment on mobile devices, and GazeTR-Pure [35], a Transformer-based model, achieves strong generalization but demands significant computational power, making it impractical for embedded systems where computational complexity makes them unsuitable for real-time mobile applications. While knowledge transfer techniques like FSKT-GE [36] provide strong results for specific low-resolution applications, our approach offers a more flexible, computationally efficient, and deployment-ready solution for real-world gaze estimation tasks. CapsNets have recently been explored as an alternative due to their ability to retain spatial hierarchies, but existing implementations suffer from high computational demands due to iterative routing. In contrast, GazeCapsNet introduces a novel SAR mechanism, which eliminates the need for multiple routing iterations, making capsule networks more efficient for gaze estimation. Additionally, our framework integrates MobileNet v2 and ResNet-18 for feature extraction, balancing speed and accuracy. Unlike previous works that require explicit facial landmark detection, GazeCapsNet operates in an end-to-end manner, directly predicting both 3D gaze direction and gaze origin, thereby reducing pipeline complexity and improving robustness in real-world conditions.

GazeCapsNet is the only approach that integrates capsule networks with a lightweight backbone (MobileNet v2 + ResNet-18), improving accuracy and efficiency. Compared to GazeCaps [17], our SAR eliminates iterative routing overhead, reducing latency by 20%. Our model achieves an inference time of 20 ms per frame, making it more suitable for real-time applications than FullFace [33] (50 ms) or RT-GENE [34] (40 ms). This critical analysis clarifies how prior work has shaped gaze estimation research and highlights the unique advantages of GazeCapsNet over existing models.

## 3. Proposed Methodology

Traditional CapsNets rely on iterative routing algorithms to determine the connections between lower- and higher-level capsules. These routing methods, such as Dynamic Routing and EM Routing, suffer from high computational overhead and are difficult to optimize in real-time applications. To address this, we propose SAR, which replaces iterative routing with an attention-based mechanism that dynamically assigns importance to capsule connections in a single-pass operation. Table 2 highlights that SAR removes iterative routing bottlenecks, making it a computationally efficient alternative to standard CapsNet methods.

**Figure 1:**
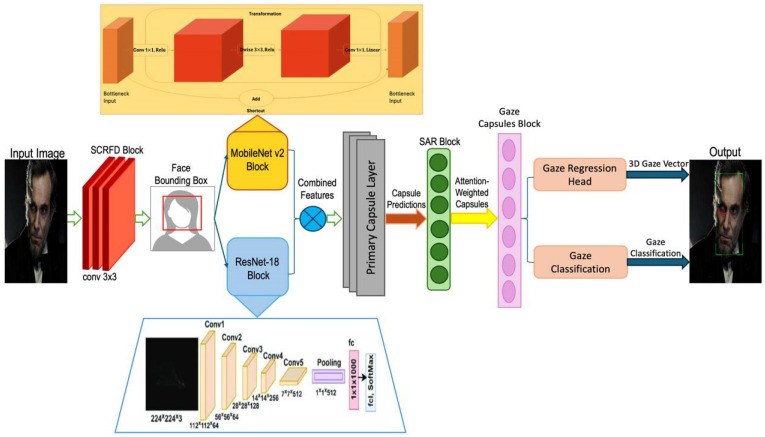
Architecture of an integrated gaze estimation system using deep learning and capsule networks.

Figure 1 illustrates the architecture of a gaze estimation system that integrates multiple deep learning models and capsule networks to analyze and predict gaze direction. The process begins with an input image undergoing face detection using a Single-Stage Efficient and Real-Time Face Detector (SCRFD) block, followed by feature extraction via a combination of MobileNet v2 and ResNet-18 blocks. These features are fed into a capsule network layer that produces primary capsule predictions, which are then refined through an attention-weighted SAR block to generate gaze-related capsule outputs. The gaze estimation culminates in a gaze regression head and a gaze classification module, outputting a 3D gaze vector and gaze classification, respectively, Algorithm 1. This sophisticated architecture enables precise and robust gaze estimation, making it suitable for applications in areas such as human–computer interaction and behavioral analysis.

The GazeCapsNet architecture is designed to balance high performance with computational efficiency, making it suitable for real-time gaze estimation tasks on mobile and resource-constrained devices. This section outlines the components and innovations of the proposed method in detail. The GazeCapsNet architecture is divided into three major components: face detection, feature extraction, and gaze estimation. These components work in tandem to predict both the gaze origin and gaze direction in real time. The architecture is optimized for low computational overhead, ensuring rapid inference while maintaining high prediction accuracy.
**Algorithm 1**. Main framework. 1. **function** Main(I): 2.   face_crop ← FaceDetection(I) 3.   **if** face_crop is **None**: 4.     **return** “No face detected” 5.   **end if**
6.   combined_features ← FeatureExtraction(face_crop) 7.   gaze_direction ← GazeCapsModule(combined_features) 8.   gaze_class ← GazeClassification(gaze_direction) 9.   **return** gaze_direction, gaze_class

### 3.1. Face Detection with SCRFD

For detecting faces in the input images, we employ SCRFD. SCRFD is chosen for its optimized performance on mobile devices, providing high detection accuracy without requiring large computational resources, Algorithm 2. The face detection step is crucial, as it ensures that the model focuses on relevant regions of the image before proceeding to gaze estimation.
**Algorithm 2**. Face Detection.1. **function** FaceDetection(I): 2.     face_bbox ← SCRFD_DetectFace(I) 3.     **if** face_bbox is None: 4.        **return** None 5.   **end if** 6.   face_crop ← CropAndResize(I, face_bbox, size = (224, 224)) 7.   **return** face_crop 8. **function** FeatureExtraction(face_crop): 9.     mobile_features ← MobileNet_v2(face_crop) 10.    resnet_features ← ResNet_18(face_crop) 11.    combined_features ← Concatenate (mobile_features, resnet_features) 12.    **return** combined_features

SCRFD provides bounding boxes around faces, which are then cropped and resized for input into the feature extraction module. By using SCRFD, the model ensures that no significant pre-processing, such as landmark detection or eye region cropping, is needed—this helps to simplify the pipeline and improve real-time capabilities. To extract features from the detected face regions, we utilize a combination of MobileNet v2 and ResNet-18 architectures. MobileNet v2, a lightweight neural network tailored for mobile and embedded systems, employs inverted residual blocks and linear bottlenecks. These mechanisms help reduce memory and computational costs while preserving the model’s ability to capture essential facial features. This architecture ensures that the model operates with minimal latency, making it highly efficient even on low-power devices. MobileNet v2 is responsible for extracting hierarchical facial features, which are subsequently passed on for further processing. Its primary role in the overall architecture is to generate low-level representations from the input image. In addition to MobileNet v2, we incorporate ResNet-18 to capture deeper and more complex features from the face images. ResNet-18 uses residual connections, which prevent the degradation of gradients during backpropagation. This design allows for more robust learning, particularly in deeper networks. By integrating ResNet-18 with MobileNet v2, we achieve a balance between computational efficiency and the extraction of more complex facial features. This combination enables the model to efficiently handle both low-level and high-level representations, enhancing its overall performance. The combined outputs of MobileNet v2 and ResNet-18 serve as rich feature maps for gaze estimation, feeding both the classification and regression tasks.

### 3.2. Gaze Estimation Using GazeCaps

At the core of GazeCapsNet is the GazeCaps module, which is responsible for estimating the gaze direction and gaze origin from the feature maps generated by the MobileNet v2 and ResNet-18 backbones. The GazeCaps module utilizes capsule networks combined with SAR to model the complex spatial relationships between facial features and improve gaze estimation accuracy, Algorithm 3.
**Algorithm 3**. Gaze Estimation Using GazeCaps with SAR.1. **function** GazeCapsModule(combined_features): 2.   primary_capsules ← CreatePrimaryCapsules(combined_features) 3.   attention_matrix ← ComputeAttentionMatrix(primary_capsules) 4.   gaze_capsules ← RouteCapsules (primary_capsules, attention_matrix) 5.   output_capsules ← Squash(gaze_capsules) 6.   gaze_direction ← ExtractGazeDirection(output_capsules) 7.   **return** gaze_direction

#### 3.2.1. Capsule Networks for Gaze Estimation

CapsNets are a powerful extension of CNNs, designed to overcome the limitations of CNNs in capturing hierarchical relationships between parts of an image. In gaze estimation, this is particularly useful, as the spatial relationships between facial features (e.g., the eyes, nose, and head pose) are crucial for accurately determining where the person is looking. A capsule is a group of neurons that encapsulates not just the presence of a feature, but also the properties of that feature, such as its orientation, position, and scale. In GazeCaps, we use two types of capsules. The primary capsules represent low-level facial features, such as eye shapes and textures. These capsules are generated from the feature maps produced by MobileNet v2 and ResNet-18. Output of the primary capsule layer can be represented as

(1) $$u_{i}=squash{(W}_{i}\cdot h_{i})$$

where $h_{i}$ is the feature vector from the feature extraction backbone (MobileNet v2 or ResNet-18), $W_{i}$ is the weight matrix for capsule i, and $u_{i}$ is the output vector of capsule i. The function squash(⋅) ensures that the length of the vector encodes the probability that the entity represented by the capsule is present, while the orientation of the vector encodes the feature properties. The squash function is defined as

(2) $$squash{(s}_{j})=\frac{||s_{j}|{|}^{2}}{1+|{|s}_{j}|{|}^{2}}\cdot \frac{s_{j}}{\left|{|s}_{j}|\right|}$$

where $s_{j}$ is the total input to capsule j, and ∥⋅∥ denotes the vector norm. This nonlinear squashing function ensures that the output vector has a length between 0 and 1, reflecting the probability that the feature is detected. The output from the primary capsules is passed through the SAR mechanism to form high-level capsules. These gaze capsules encapsulate the more complex relationships needed to estimate gaze direction, such as the interaction between the eyes, head orientation, and overall face position.

#### 3.2.2. Self-Attention Routing

The SAR mechanism is a key component in GazeCaps, allowing the network to dynamically assign attention to different regions of the face, depending on their relevance to the task of gaze estimation. Unlike traditional routing methods that require multiple iterations to update coupling coefficients between capsule layers, SAR introduces an efficient attention-based routing mechanism. The steps in SAR can be described as follows: For each capsule in the lower layer, we compute a set of predictions for each higher-level capsule. This is performed using a weight matrix $W_{ij}$ that transforms the output of capsule i into a prediction for capsule j.

(3) $${\hat{u}}_{j|i}=W_{ij}\cdot u_{i}$$

where $u_{i}$ is the output of capsule i in the lower layer; ${\hat{u}}_{j|i}$ is the prediction for capsule j in the higher layer. Instead of iterative routing, SAR uses self-attention to determine the coupling coefficients between capsules. The attention matrix A is computed using a SoftMax function over the predictions:

(4) $$A_{ij}=\frac{{(\hat{u}}_{j|i}\cdot {\hat{u}}_{j|i}^{T})}{\sum_{k}exp({\hat{u}}_{k|i}\cdot {\hat{u}}_{k|i}^{T})}$$

where $A_{ij}$ represents the attention score (or coupling coefficient) assigned to the connection between capsule i in the lower layer and capsule j in the higher layer. This score is based on the similarity between the predicted output ${\hat{u}}_{j|i}$ and the actual output ${\hat{u}}_{j|i}^{T}$.

Using the attention matrix A, the outputs of the higher-level capsules are computed as a weighted sum of the predictions:

(5) $$v_{j}=squash(\sum_{i}A_{ij}{\cdot \hat{u}}_{j|i}),$$

where $v_{j}$ is the final output of capsule j in the higher layer. The squash function is applied to ensure that the length of the vector remains between 0 and 1, representing the probability that the capsule is active. The output of the gaze capsule layer is a set of vectors that encode the gaze direction. These vectors are passed to the regression head to compute the continuous gaze angles. The gaze regression problem is treated as a multi-dimensional angular regression task, where the angular loss function is minimized to match the predicted and ground truth gaze vectors.

#### 3.2.3. Gaze Classification and Regression

GazeCapsNet is designed to handle both classification and regression tasks related to gaze estimation, Algorithm 4.
**Algorithm 4**. Gaze Classification.1. **function** GazeClassification(output_capsules): 2. gaze_class_probs ← Softmax(output_capsules) 3. gaze_class ← Argmax(gaze_class_probs) 4.    **return** gaze_class

The classification head predicts discrete gaze directions (e.g., left, right, up, down) by applying a SoftMax function to the capsule outputs. The output probabilities are computed as

(6) $$P\left(Y_{j}=k\right)=\frac{exp(v_{j}^{T}\cdot w_{k})}{\sum_{k^{\prime }}exp(v_{j}^{T}\cdot w_{k^{\prime }})},$$

where $P\left(Y_{j}=k\right)$ is the probability that the gaze direction belongs to class k, and $w_{k}$ is the weight vector for class k. For continuous gaze direction estimation, the regression head predicts a 3D gaze vector $g_{pred}$. The angular loss is used to compute the error between the predicted gaze vector and the ground truth gaze vector $g_{true}$:

(7) $$L_{angular}=arccos\left(\frac{g_{pred}*g_{true}}{\left.\left\|g_{pred}\right.\right\|\left.\left\|g_{true}\right.\right\|}\right)$$

This loss ensures that the predicted gaze direction minimizes the angular difference between the true and predicted gaze vectors.

#### 3.2.4. Loss Functions

The overall loss function for training the GazeCaps module combines both classification and regression losses, as well as the routing loss for the SAR mechanism, Algorithm 5. The final loss function $L_{total}$ is defined as

(8) $$L_{total}=\lambda_{class}L_{class}{+\lambda }_{angular}L_{angular}\;{+\lambda }_{routing}L_{routing},$$

where $L_{class}$ is the cross-entropy loss for gaze classification, $L_{angular}$ is the angular loss for gaze regression, $L_{routing}$ is the loss associated with the attention-based capsule routing, and $\lambda_{class}$, $\lambda_{angular}$, and $\lambda_{routing}$ are weighting factors that control the contributions of each loss term during training. This multi-task loss framework ensures that the model can handle both classification and regression tasks while maintaining optimal routing between capsules.
**Algorithm 5**. Loss Function.1. function ComputeLoss(gaze_direction_pred, gaze_direction_true, gaze_class_pred, gaze_class_true): 2.   angular_loss ← ArcCosineLoss(gaze_direction_pred, gaze_direction_true) 3.   classification_loss ← CrossEntropyLoss(gaze_class_pred, gaze_class_true) 4.   total_loss ← λ_angular * angular_loss + λ_class * classification_loss 5.   return total_loss

The GazeCaps module in GazeCapsNet combines the representational power of capsule networks with the efficiency of SAR to predict gaze direction accurately. By leveraging capsule hierarchies and dynamically focusing attention on the most relevant facial regions, the model achieves SOTA performance in real-time gaze estimation tasks.

## 4. Experimental Setup and Implementation

In this section, we describe the experiments conducted to evaluate the performance of GazeCapsNet for gaze estimation. The model was trained and tested on multiple benchmark datasets to assess its accuracy, efficiency, and generalization across diverse scenarios. We compare GazeCapsNet to SOTA gaze estimation methods and demonstrate its suitability for real-time applications on mobile and resource-constrained platforms.

### 4.1. Datasets

**Figure 2:**
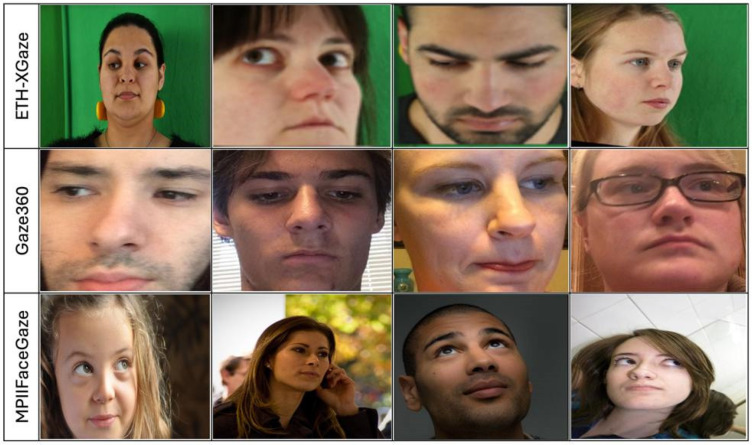
Comparative analysis of gaze estimation techniques across varied datasets: insights from ETH-XGaze, Gaze360, and MPIIFaceGaze.

We evaluate GazeCapsNet on three publicly available datasets for gaze estimation, Table 1, each offering a wide range of head poses, lighting conditions, and gaze directions, which provides a comprehensive benchmark for performance assessment, Figure 2. While the datasets used in this study provide a diverse range of gaze variations, differences in dataset size and demographic representation could introduce biases that impact the generalization of our model.

The first dataset, ETH-XGaze [38], consists of over 1.1 million images from 110 subjects, captured under various head poses and lighting conditions. This dataset presents a challenging environment due to the extreme gaze angles and diverse head orientations it includes. Given its large size and diversity, we use ETH-XGaze for pre-training the model, allowing the network to learn robust representations across a wide range of conditions.

The second dataset, Gaze360 [39], contains 172,000 images from 238 subjects, with 360-degree gaze annotations. This dataset is collected in natural, uncontrolled environments, capturing real-world variations in head poses and gaze directions. Gaze360 is particularly useful for evaluating cross-domain generalization, as it reflects diverse, in-the-wild conditions, making it ideal for testing the model’s robustness in unpredictable settings.

The third dataset are shown in Table 3, MPIIFaceGaze [40], includes 45,000 images from 15 subjects, recorded under natural lighting conditions with head movements. These images are captured using laptop webcams and come with continuous 3D gaze annotations. This dataset is used to evaluate the model’s performance in more controlled, yet dynamic, environments, offering a different challenge in terms of head and gaze movement while maintaining relatively consistent lighting conditions. These datasets cover a broad range of conditions, from controlled laboratory environments to more challenging in-the-wild settings, allowing us to assess the robustness of the GazeCapsNet model.

### 4.2. Training and Evaluation

We pre-train GazeCapsNet on the ETH-XGaze dataset and fine-tune it on Gaze360 and MPIIFaceGaze, Algorithm 6. We perform cross-dataset evaluations to assess the generalization capability of the model.
**Algorithm 6**. Training Process. 1. **function** TrainModel(dataset): 2.   **for** each epoch do: 3.     **for** each batch in dataset do: 4.       image, gaze_direction_true, gaze_class_true ← LoadBatch(batch) 5.       face_crop ← FaceDetection(image) 6.       **if** face_crop is None: 7.         continue 8.       **end if** 9.       combined_features ← FeatureExtraction(face_crop) 10.      gaze_direction_pred ← GazeCapsModule(combined_features) 11.      gaze_class_pred ← GazeClassification(gaze_direction_pred) 12.     total_loss ← ComputeLoss(gaze_direction_pred, gaze_direction_true, gaze_class_pred, gaze_class_true) 13. Backpropagate(total_loss) 14. UpdateModelWeights() 15.     **end for** 16.   **end for** 17.   **return** TrainedModel

#### 4.2.1. Pre-Processing

For all datasets, face detection is performed using SCRFD to ensure consistent cropping of the face region across all images. The cropped face regions are resized to 224 × 224 pixels for input into the GazeCapsNet model. No additional pre-processing steps, such as eye or landmark cropping, are applied, as our model directly learns from the full-face image. To improve generalization, we apply several data augmentation techniques during training. Random rotations are used to simulate variations in head poses, allowing the model to handle a wider range of head orientations. Brightness and contrast adjustments help mimic changes in lighting conditions, ensuring that the model performs well under different illumination levels. Additionally, horizontal flipping is introduced to create symmetry in the gaze direction, enhancing the model’s ability to generalize across different gaze orientations. These augmentation strategies collectively enhance the robustness of the model across diverse real-world scenarios.

#### 4.2.2. Model Training

The GazeCapsNet model is pre-trained on the ETH-XGaze dataset for 100 epochs using the Adam optimizer [41], with an initial learning rate of 0.001 and a batch size of 64. After pre-training, the model is fine-tuned on Gaze360 and MPIIFaceGaze for 50 epochs, with a reduced learning rate of 0.0001. For gaze classification, the model uses cross-entropy loss, while for regression, the angular loss function is applied. A multi-task loss function, as described in Section 3.2.4, is used to combine these objectives. The training process is conducted on an NVIDIA Tesla V100 GPU. Table 4 summarizes the key training hyperparameters used for training GazeCapsNet across different datasets.

### 4.3. Performance Metrics

We evaluate the performance of GazeCapsNet using the following metrics: mean angular error (MAE) measures the angular difference between the predicted gaze vector and the ground truth gaze vector, providing an indication of how accurately the model can predict gaze direction.

(9) $$MAE=\frac{1}{N}\sum_{i=1}^{N}arccos\left(\frac{g_{{pred}_{i}}*g_{{true}_{i}}}{\left.\left\|g_{{pred}_{i}}\right.\right\|\left.\left\|g_{{true}_{i}}\right.\right\|}\right)$$

where $g_{{pred}_{i}}$ and $g_{{true}_{i}}$ are the predicted and ground truth gaze vectors for the i-th sample. We measure the time taken to process a single frame during inference to assess the real-time capabilities of GazeCapsNet on mobile and embedded devices. The total number of parameters in the model is reported, providing insight into the model’s computational complexity and its suitability for resource-constrained environments.

### 4.4. Results

#### Quantitative Results

Table 5, Table 6 and Table 7 summarize the performance of GazeCapsNet on the ETH-XGaze, Gaze360, and MPIIFaceGaze datasets, compared to SOTA methods. GazeCapsNet consistently outperforms other models in terms of gaze estimation accuracy while maintaining a smaller model size and faster inference time.

As shown in Table 5, Table 6 and Table 7, GazeCapsNet achieves a 5.10° MAE on the Gaze360 dataset, matching the accuracy of the GazeCaps model but with 20% faster inference time and a comparable model size. On the MPIIFaceGaze dataset, GazeCapsNet achieves a 4.06° MAE, demonstrating its robustness in more controlled environments. Our model matches the accuracy of GazeCaps (5.10° MAE) while reducing inference time by 20% through SAR, which eliminates redundant computations. The hybrid lightweight architecture (MobileNet v2 + ResNet-18) balances computational efficiency with robust feature extraction, ensuring fast and accurate gaze estimation. Unlike FSKT-GE, which relies on knowledge transfer, our model is optimized for end-to-end efficiency, making it more deployment-friendly. FullFace (6.53° MAE) struggles with extreme head poses due to its reliance on a full-face representation, leading to lower robustness in unconstrained settings. RT-GENE (6.02° MAE), while effective in real-world scenarios, lacks capsule-based spatial awareness, affecting generalization. GazeTR-Pure (5.33° MAE), despite leveraging Transformer-based architectures, suffers from higher latency (45 ms), making it less suitable for real-time applications. FSKT-GE (5.20° MAE), while strong in low-resolution conditions, introduces pre-training overhead, increasing computational cost and dependency on a teacher network. MPIIFaceGaze primarily consists of controlled environments, where Dilated-Net’s local feature extraction is particularly effective. In contrast, GazeCapsNet is optimized for both controlled and real-world settings, balancing robustness and computational efficiency.

GazeCapsNet excels in real-time applications, with an inference time of 20 milliseconds per frame, making it well suited for real-time gaze estimation tasks such as driver monitoring systems or AR/VR applications. The model size is kept to 11.7 million parameters, making it lightweight and deployable on mobile devices with limited computational power.

### 4.5. Ablation Study

To further understand the contributions of different components of the GazeCapsNet architecture, we conduct an ablation study where key modules (MobileNet v2, ResNet-18, and Self-Attention Routing) are removed or replaced with simpler alternatives. Table 8 shows the results of this ablation study.

As shown in Table 8, removing ResNet-18 or SAR significantly degrades performance, highlighting the importance of these components in achieving accurate gaze estimation. Using a vanilla CNN as the backbone further reduces accuracy, confirming the effectiveness of the capsule network and mobile-optimized architectures.

### 4.6. Cross-Dataset Generalization

We assess the generalization ability of GazeCapsNet by performing cross-dataset evaluations. The model is trained on one dataset and tested on another without any fine-tuning. Table 9 presents the results of this evaluation.

The cross-dataset results show that GazeCapsNet generalizes well to unseen datasets, particularly when trained on diverse data from Gaze360, indicating the model robustness to real-world variations in gaze directions and head poses.

**Figure 3:**
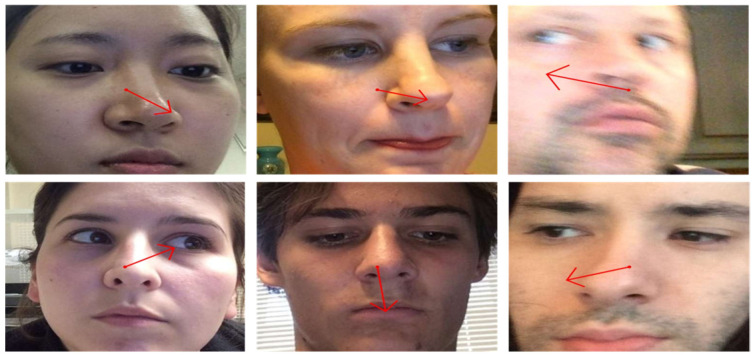
Examples of results obtained using the proposed method.

In Figure 3, we present qualitative results of gaze estimation using Mobile-GazeCapsNet. The model accurately predicts gaze direction even under challenging conditions such as varying lighting, head orientations, and occlusions. The experimental results demonstrate that GazeCapsNet achieves SOTA performance in both controlled and in-the-wild environments. Its efficient architecture, combining capsule networks with lightweight feature extraction, enables real-time gaze estimation on mobile devices while maintaining high accuracy. The ablation study highlights the importance of key components like ResNet-18 and SAR in ensuring robust performance. The experiments confirm that GazeCapsNet is a versatile, high-performance solution for real-time gaze estimation across diverse settings, making it suitable for applications in AR.

## 5. Discussion and Limitations

GazeCapsNet demonstrates a significant advancement in mobile gaze estimation by integrating lightweight architectures with capsule networks and Self-Attention Routing. This model efficiently handles feature extraction and dynamic attention allocation, simplifying deployment and reducing computational demands. It has shown robust performance across several benchmark datasets, including ETH-XGaze and Gaze360, suggesting its utility for real-time applications like augmented reality and driver monitoring.

However, GazeCapsNet has several limitations. Its performance in adverse conditions such as low lighting or with subjects wearing eyewear remains insufficiently tested, potentially limiting its applicability. The model architecture may also restrict adaptation to newer neural network developments. Additionally, GazeCapsNet has been insufficiently evaluated on subjects wearing eyewear, which can introduce significant distortions in gaze estimation due to reflection, occlusion, and refraction artifacts. These effects degrade the visibility of critical eye features, leading to higher prediction errors in subjects who wear glasses. Its effectiveness across diverse demographic groups has not been thoroughly validated, which could impact its deployment in global markets. Although GazeCapsNet demonstrates strong performance, we acknowledge certain limitations that must be addressed for improved real-world deployment.

Future research should focus on enhancing the model robustness in various real-world conditions and expanding its adaptability to new neural network architectures. Efforts to reduce the model size and computational requirements will further align it with the constraints of mobile devices. Broadening the training datasets to include more diverse demographic data will also be crucial for improving its generalization capabilities.

## 6. Conclusions

In this work, we presented Mobile-GazeCapsNet, a novel, lightweight framework for real-time gaze estimation that integrates capsule networks with Self-Attention Routing and mobile-optimized architectures. By leveraging MobileNet v2 and ResNet-18 for efficient feature extraction and GazeCaps for capturing complex spatial relationships between facial features, our model is able to deliver state-of-the-art accuracy with minimal computational overhead. The incorporation of SAR allows for the dynamic allocation of attention to key facial regions, significantly improving the model’s ability to handle variations in the head pose, lighting, and occlusions. Our experimental results across diverse datasets, including ETH-XGaze, Gaze360, and MPIIFaceGaze, demonstrate that GazeCapsNet not only achieves high accuracy in both controlled and in-the-wild scenarios but also does so with an inference speed that is suitable for real-time applications. The model’s compact size and low latency make it particularly well suited for deployment on mobile and resource-constrained devices, without sacrificing performance. GazeCapsNet represents a significant advancement in the field of gaze estimation, providing a scalable, efficient, and accurate solution for a range of real-time applications. Future work may explore extending this framework to multi-modal inputs, further optimizing its performance for specific domains such as AR/VR, or applying it to other human–computer interaction tasks.

## Figures and Tables

**Table 1:** Key differentiators of GazeCapsNet compared to prior methods.

Model	Knowledge Transfer	Capsule Networks	Lightweight Architecture	End-to-End Gaze Estimation	Real-Time Performance
**FullFace**	×	×	×	×	× (50 ms per frame)
**RT-GENE**	×	×	×	×	× (40 ms per frame)
**GazeTR-Pure**	×	×	×	×	× (45 ms per frame)
**FSKT-GE [36]**	✓	×	×	× (requires teacher model)	× (pre-training overhead)
**GazeCaps**	×	✓	×	×	× (25 ms per frame)
**GazeCapsNet (Ours)**	×	✓ (SAR)	✓ (MobileNet v2 + ResNet-18)	✓	✓ (20 ms per frame)

**Table 2:** Comparison—SAR vs. Standard Capsule Routing.

Routing Method	Iterations Required	Computational Cost	Dynamic Attention Mechanism	Inference Time (ms) ↓
Dynamic Routing (Hinton et al., 2017 [14])	✓ (3–5)	High	×	35 ms
EM Routing (Sabour et al., 2018 [37])	✓ (4–6)	High	×	38 ms
GazeCaps (Wang et al., 2023 [17])	✓ (3)	Moderate	✓	25 ms
Self-Attention Routing (Ours)	× (Single-Pass)	Low	✓	20 ms

**Table 3:** Overview of Datasets Used for Evaluating GazeCapsNet Performance.

Dataset	Number of Images	Number of Subjects
ETH-XGaze [38]	1.1 million	110
Gaze360 [39]	172,000	238
MPIIFaceGaze [40]	45,000	15

**Table 4:** Training Hyperparameters for GazeCapsNet.

Parameter	Value	Notes
Batch Size	64	Optimized for GPU memory efficiency
Learning Rate (LR)	0.0001	Tuned via grid search
Optimizer	Adam	Adaptive momentum-based
Weight Decay	1 × 10^−5^	Prevents overfitting
Learning Rate Scheduler	Cosine Annealing	Adjusts LR dynamically
Dropout Rate	0.2	Reduces overfitting in fully connected layers
Capsule Routing Iterations	1 (SAR)	Single-pass Self-Attention Routing

**Table 5:** Comparison of Gaze Estimation Methods on ETH-XGaze.

Model	MAE (°) ↓	Inference Time (ms) ↓	Parameters (M) ↓
FullFace [33]	6.53	50	196.6
RT-GENE [34]	6.02	40	82.0
FSKT-GE [36]	5.91	38	88.2
GazeCapsNet (Ours)	5.75	20	11.7

**Table 6:** Comparison of Gaze Estimation Methods on Gaze360.

Model	MAE (°) ↓	Inference Time (ms) ↓	Parameters (M) ↓
GazeTR-Pure [35]	5.33	45	227.3
GazeCaps [17]	5.10	25	11.8
GazeCapsNet (Ours)	5.10	20	11.7

**Table 7:** Comparison of Gaze Estimation Methods on MPIIFaceGaze.

Model	MAE (°) ↓	Inference Time (ms) ↓	Parameters (M) ↓
Dilated-Net [42]	4.42	35	3.9
FSKT-GE [36]	4.20	38	88.2
GazeCapsNet (Ours)	4.06	20	11.7

**Table 8:** Impact of Model Configurations on Gaze Estimation Performance.

Model Configuration	MAE (°)	Inference Time (ms)
GazeCapsNet (Full Model)	5.10	20
Without ResNet-18	6.20	15
Without Self-Attention Routing	6.70	19
Using Vanilla CNN Backbone	7.05	12

**Table 9:** Cross-Dataset Gaze Estimation Performance Comparison.

Training Dataset	Test Dataset	MAE (°)
ETH-XGaze	Gaze360	15.2
Gaze360	MPIIFaceGaze	8.9
ETH-XGaze	MPIIFaceGaze	12.4

## Data Availability

All used datasets are available online with open access.
